# Physiological Peculiarities of Lignin-Modifying Enzyme Production by the White-Rot Basidiomycete *Coriolopsis gallica* Strain BCC 142

**DOI:** 10.3390/microorganisms5040073

**Published:** 2017-11-17

**Authors:** Vladimir Elisashvili, Eva Kachlishvili, Mikheil D. Asatiani, Ramona Darlington, Katarzyna H. Kucharzyk

**Affiliations:** 1 Agricultural University of Georgia, 240 David Agmashenebeli Alley, 0159 Tbilisi, Georgia; e.kachlishvili@agruni.edu.ge (E.K.); m.asatiani@agruni.edu.ge (M.D.A.); 2 Battelle Memorial Institute, 505 King Ave, Columbus, OH 43212, USA; darlingtonr@battelle.org (R.D.); kucharzyk@battelle.org (K.H.K.)

**Keywords:** *Coriolopsis gallica*, white-rot basidiomycetes, lignin-modifying enzymes, production, optimization, submerged fermentation

## Abstract

Sixteen white-rot Basidiomycota isolates were screened for production of lignin-modifying enzymes (LME) in glycerol- and mandarin peel-containing media. In the synthetic medium, *Cerrena unicolor* strains were the only high laccase (Lac) (3.2–9.4 U/mL) and manganese peroxidase (MnP) (0.56–1.64 U/mL) producers while one isolate *Coriolopsis gallica* was the only lignin peroxidase (LiP) (0.07 U/mL) producer. Addition of mandarin peels to the synthetic medium promoted Lac production either due to an increase in fungal biomass (*Funalia trogii*, *Trametes hirsuta*, and *T. versicolor*) or enhancement of enzyme production (*C. unicolor*, *Merulius tremellosus*, *Phlebia radiata*, *Trametes ochracea*). Mandarin peels favored enhanced MnP and LiP secretion by the majority of the tested fungi. The ability of LiP activity production by *C. gallica*, *C. unicolor*, *F. trogii*, *T. ochracea*, and *T. zonatus* in the medium containing mandarin-peels was reported for the first time. Several factors, such as supplementation of the nutrient medium with a variety of lignocellulosic materials, nitrogen source or surfactant (Tween 80, Triton X-100) significantly influenced production of LME by a novel strain of *C. gallica*. Moreover, *C. gallica* was found to be a promising LME producer with a potential for an easy scale up cultivation in a bioreactor and high enzyme yields (Lac-9.4 U/mL, MnP-0.31 U/mL, LiP-0.45 U/mL).

## 1. Introduction

Lignin, next to cellulose and hemicellulose polymer, represents one of the most abundant renewable organic materials and its biodegradation plays a key role in the carbon cycling. Lignin is mainly decomposed by white-rot basidiomycetes (WRB) in aerobic conditions and the presence of one or more extracellular oxidative lignin-modifying enzymes (LME) such as lignin peroxidase (LiP, EC 1.11.1.14), manganese peroxidase (MnP, EC 1.11.1.13), versatile peroxidase (EC 1.11.1.16) and laccase (EC 1.10.3.2) is essential for lignin degradation [1,2,3].

Besides their fundamental importance in lignin degradation, LME are utilized in various biotechnological processes and applications ranging from pulp and paper, food, textile, dye and cosmetics industries to sustainable production of renewable chemicals, materials, fuels, organic synthesis and bioremediation [3,4]. With an increase in supply and demand for LME, their cost, yield and production efficiency for industrial and environmental applications need to be improved. Thus, several strategies such as search and bioengineering of new LME producing fungal species, exploitation of cheap raw plant materials for growth substrates, optimization of nutrient media and cultivation conditions, usage of effective inducers, surfactants, and development of better bioprocess technologies [5,6,7] have been tested to date. These studies clearly indicated that culture conditions affect fungal physiology and expression of LME. Some of the specific factors affecting LME synthesis and the relative amounts of individual enzymes may include composition and concentration of carbon source, phenolic and aromatic compounds, microelements, mode of WRB cultivation and aeration.

In the past few years, a number of WRB have been screened to identify promising producers of LME, while a few fungal species have demonstrated encouraging production of laccase [8,9,10,11] and lignin-modifying peroxidases [5,7,12,13]. Among a variety of lignin-degrading fungi, *Phanerochaete chrysosporium* was one of the most comprehensively studied species to elucidate LiP and MnP production mechanisms [2,6,7,12,13,14,15,16]. Other WRB from different taxonomic groups have been evaluated, but more work needs to be performed to identify key species and to determine nutrient and cultivation conditions for efficient production and high yield of LME. Therefore, an efficient production system with affordable basal medium is critical for future scale up.

The aims of this study were to comparatively assess the ability of several new Georgian WRB and well-known LME producers for efficient secretion of the LME in submerged cultivation on cheap synthetic and lignocellulose-containing media and to elucidate the nutritional requirements and growth conditions providing maximum production of LME by a novel enzyme producer, *Coriolopsis gallica*.

## 2. Materials and Methods

### 2.1. Organisms and Inocula Preparation 

White-rot fungi from basidiomycetes culture collection of the Agricultural University of Georgia were used in this study: *Cerrena unicolor* strains BCC 300, BCC 301, BCC 302, and BCC 303, *Coriolopsis gallica* BCC 142, *Funalia trogii* BCC 146, *Merulius tremellosus* BCC 206, *Phanerochaete chrysosporium* BCC 1309, *Trametes hirsuta* BCC 119, *T. ochracea* BCC 1009, *T. versicolor* strains BCC 113, BCC 159, and *T. zonatus* BCC 540. The strains of *P. chrysosporium* ATCC (American Type Culture Collection) 24725, *P. chrysosporium* ATCC 34541, and *Phlebia radiata* ATCC 64658 were purchased from the ATCC. The fungal inocula were prepared by growing the fungal mycelium from agar slants on a rotary shaker in 250 mL flasks containing 100 mL of the medium (per L): 15 g glucose, 3 g peptone, 1 g KH_2_PO_4_, 0.5 g MgSO_4_·7H_2_O, 3 g yeast extract. Cultures were incubated at 27 °C and 150 rpm for 7 days. The final fungal biomass was homogenized in a Waring laboratory blender and used as an inoculum for shake-flask cultures.

### 2.2. Lignocellulosic Materials

The following available in large amounts in Georgia plant raw materials were tested as growth substrates in order to establish their impact to *C. gallica* 142 enzyme activity: residue after ethanol production from the wheat grains (EPR, contains 43% extractives, 7% cellulose, 34% crude protein), wheat bran (56% extractives, 8% cellulose, 6% lignin, 15% crude protein), sunflower oil cake (SOC, 25% extractives, 6% cellulose, 36% crude protein, 0.11% Cu), mandarin peels (67% extractives, 24% cellulose, 7% crude protein), walnut pericarp (52% extractives, 16% cellulose, 7% lignin, 10% crude protein), and banana peels (47% extractives, 18% cellulose, 10% lignin, 8% crude protein). All plant residues were oven-dried at 50 °C and ground to powder in a laboratory mill KM-1500 (MRC, Holon, Israel) prior to addition to the nutrient medium. The contents of water-soluble extractives were determined gravimetrically after suspension of these materials in water (10%, *w*/*v*) and autoclaving at 115 °C for 30 min. The total nitrogen was determined according to Kjeldahl method with Nessler reactive after pre-boiling of samples in 0.5% solutions of trichloroacetic acid for 15 min to remove non-protein content. True protein content was calculated as the total nitrogen multiplied by 4.38. Cellulose and lignin in samples were determined by the method of Updegraff [17] and the gravimetric method with 72% sulfuric acid, respectively.

### 2.3. Shake-Flask Cultivation Conditions

The submerged fungal cultivation was conducted in Innova 44 shaker (New Brunswick Scientific, Edison, NJ, USA) at 150 rpm. Cultivation temperature of *P. chrysosporium* was 37 °C while other fungi were grown at 27 °C. Homogenized mycelium (3 mL) was used to inoculate 50 mL of synthetic medium containing (per L): 10 g glycerol, 2 g peptone; 2 g yeast extract, 0.1 g CaCl_2_, and 0.005 g FeSO_4_.

To study the effect of lignocellulosic materials on production of LME, 20 g/L of the above-mentioned plant residues were supplemented to the synthetic medium as additional growth substrates.

To evaluate the effect of aromatic compounds on the enzyme production 0.5 mM (mol/L) of xylidine, veratryl alcohol, 2,6-dimethoxyphenol, pyrogallol, vanillic acid, guaiacol and 0.3 mM (mol/L) of trinitrotoluene (TNT) and hydroquinone were added into the control basal medium containing 40 g/L mandarin peels, prior to inoculation. To accelerate enzyme secretion, three known surfactants: Tween 80, polyethylene glycol (PEG) and Triton X-100 were added to the cultures. All chemicals used were of analytical grade and purchased from Sigma-Fluka-Aldrich (St. Luis, MO, USA).

The pH of all media was adjusted to 5.0 prior to sterilization and all submerged culture experiments were carried for 14–17 days. At predetermined time intervals, 1 mL of culture was sampled and solids were separated by centrifugation (Eppendorf 5417R, Hamburg, Germany) at 10,000× *g* for 5 min at 4 °C. The supernatants were analyzed for pH, reducing sugars and enzyme activities.

All experiments were performed twice using three replicates at each time point. All results were expressed as the mean ± SD with only *p* ≤ 0.05 considered as statistically significant.

### 2.4. Cultivation in Bioreactor

To scale up the *C. gallica* LiP production, cultivation was performed in the 7 L fermenter LILFUS GX (Incheon, South Korea) with three Rushton impellers. The fermenter was filled with 5 L of the optimized medium (per L): 40 g mandarin peels, 5 g glycerol; 1 g KH_2_PO_4_, 2 g peptone, 2 g yeast extract, 0.5 mM pyrogallol, 0.5 g MgSO_4_, 0.1 g CaCl_2_, 3 mL polypropylene glycol 2000 and the pH was adjusted to 5.0. After 5 and 8 days of fermentation, 200 mL of distilled water was added to the fermenter to compensate evaporation. The fermenter equipped with pH, temperature and pO_2_ probes was sterilized (121 °C, 40 min) and inoculated with 500 mL of homogenized mycelium. Fermentation was carried out without baffles at 27 °C and at the constant airflow rate of 1 *v*/*v*/min. During the fermentation process, samples were collected daily and analyzed for enzyme activity. After 10 days of fermentation, fungal biomass was separated from culture liquid by successive filtration and centrifugation at 5400× *g* for 15 min. Enzyme preparation was isolated from the culture liquid by precipitation with (NH_4_)_2_SO_4_ at 70% saturation and the precipitate was dissolved in 0.05 M (mol/L) acetate buffer (pH 5.5).

### 2.5. Enzyme Activity Assys

Laccase activity was determined spectrophotometrically (Camspec M501, Cambridge, UK) at 420 nm as the rate of 0.25 mM (mol/L) ABTS (2,2′-azino-bis-(3-ethylthiazoline-6-sulfonate)) oxidation in 50 mM (mol/L) Na-acetate buffer (pH 3.8) at room temperature [18]. MnP activity was measured at 270 nm by following the formation of a Mn^3+^-malonate-complex [19] and by oxidation of Phenol Red [20] in the presence of 0.1 mM (mol/L) H_2_O_2_. LiP activity was determined spectrophotometrically at 310 nm by the rate of oxidation of 2 mM (mol/L) veratryl alcohol in 0.1 M sodium tartrate buffer (pH 3.0) with 0.2 mM (mol/L) hydrogen peroxide [21]. To establish true peroxidase activity, activities in the absence of H_2_O_2_ were subtracted from the values obtained in the presence of hydrogen peroxide. One unit (U) of LME activity was defined as the amount of enzyme that oxidized 1 μmoL of substrate per minute.

Endoglucanase (CMCase, EC 3.2.1.4) activity was determined in accordance with the IUPAC (International Union of Pure and Applied Chemistry) recommendations using 1% (*w*/*v*) carboxymethyl cellulose (sodium salt, low viscosity, Sigma, Schnelldorf, Germany) in 50 mM citrate buffer (pH 5.0) at 50 °C for 10 min [22]. Glucose standard curves were used to calculate the cellulase activity. Release of glucose was measured using the dinitrosalicylic acid reagent method [23]. One unit of CMCase activity was defined as the amount of enzyme releasing 1 μmol of glucose per minute.

## 3. Results 

### 3.1. Screening of White-Rot Basidiomycetes for Lignin-Modifying Enzyme Production

Sixteen WRB strains were screened for LME production in submerged cultivation experiments, both in synthetic and in lignocellulose-containing media. When cultivated in glycerol-based medium, all fungal strains grew in the form of small pellets, and an increase in pH (from 5.0 to 5.3–6.5) was observed (Table 1). The measurements of enzyme activity revealed a diversity of LME expression in the growth medium. As expected, no laccase activity was revealed during cultivation of *P. chrysosporium* strains while low laccase activity was detected in culture liquids of *P. radiata* and *T. ochracea*. Among the fungi of genus *Trametes, T. zonatus* 540 and *T. versicolor* 159 secreted 4220 and 2350 laccase U/L, respectively. Four *C. unicolor* strains tested produced the highest laccase activities although the number of enzyme units differed significantly from strain to strain with expression of 3190 to 9410 laccase U/L. The same *C. unicolor* strains were capable of producing the highest MnP activities in cultivation in the synthetic medium. Other WRB either showed very low MnP activities or failed to produce this enzyme in the same cultivation conditions. Moreover, with the exception of *C. gallica* 142, none of the tested WRB expressed LiP in the glycerol-containing medium.

Subsequently, selected WRB were screened for enzyme production in the same glycerol-containing medium supplemented with mandarin peels that promotes LME production by majority of the fungi studied in our group [5,9,24]. The cultivation process was accompanied by abundant fungal growth and changes in pH with final values ranging from 4.8 to 6.7 (Table 2). When laccase production was compared between strains cultivated in glycerol-containing medium as the sole carbon source to strains grown in mandarin peels-based medium, the latter proved to promote higher laccase secretion. For example, *C. unicolor* 303 and other tested strains of this species produced the highest laccase activities ranging from 16,620 U/L to 38,290 U/L. High laccase activities were also determined in cultivation of *T. zonatus* 540, *T. ochracea* 1009, *C. gallica* 142 and *P. radiata* 64658. The laccase activities for these species showed a 2- to 22-fold increase in cultivation with mandarin peels.

Production of MnP by fungal cultures was measured using two substrates: manganese (II) ions and phenol red. The enzyme activity data listed in Table 1 and Table 2 show no correlation between activity values obtained with two assays widely employed in MnP studies. It is possible that isoforms of MnP produced by the tested fungi had different affinity to the substrates used in tested assays. Based on Mn^2+^ oxidation, the highest MnP activity was detected on day 7 for *C. unicolor* 301 submerged culture (2760 U/L, 3-fold higher than that in synthetic medium). Other WRB species secreted only low or trace MnP activities. Moreover, *P. chrysosporium* also produced manganese oxidizing enzyme, but no MnP activity was detected by phenol red oxidation assay. Minor amounts of LiP were detected in all tested fungal cultures, with the exception of *M. tremellosus* 206, when grown in medium with mandarin peels. Among the fungal strains tested, *C. gallica* 142, *C. unicolor* 300 and *P. chrysosporium* 1309 produced the highest LiP activities: 210 U/L, 160 U/L and 150 U/L, in respective cultures (Table 2). On the other hand, two *P. chrysosporium* ATCC strains, well-known for LiP secretion, showed only trace amounts (<0.02 U/mL) of this enzyme activity.

### 3.2. Effect of Lignocellulosic Growth Substrates on LME Production

Determination of optimal cultivation conditions for a variety of industrially important fungal species is of high practical value. Therefore, the subsequent experiments focused on the optimization of cultivation conditions for maximum LiP production by the new strain *C. gallica* 142 recently isolated from the forest close to Tetritskaro, Georgia. A common approach in the development of fermentation technologies is selection of an appropriate plant raw materials containing significant concentrations of soluble carbohydrates and inducers for an abundant growth of fungi and efficient production of LME. In this study, several plant raw materials were tested as growth substrates in order to assess the capability of *C. gallica* 142 to produce LME (Table 3). These residues are of great interest for the microbial fermentation as growth substrates since they are rich in readily available carbohydrates, nitrogen, and microelements. Moreover, walnut pericarp and mandarin peals are especially rich in a wide spectrum of aromatic compounds [25]. The lignified wheat straw (36% extractives, 36% cellulose, 18% lignin, 4% crude protein) also was tested for comparison. The first batch of experiments focused on exploring correlation between LME production and a type of lignocellulosic growth substrates added. Submerged cultivation of *C. gallica* 142 in medium containing different lignocellulosic materials revealed the highest laccase activities when the fungus was grown with SOC (27,280 U/L) or wheat bran (19,720 U/L) (Table 3). Unexpectedly, wheat straw, the most recalcitrant growth substrate, promoted comparatively high laccase secretion. In contrast, walnut pericarp and mandarin peels were rather poor inducers of laccase with only 6490 and 4680 U/L, respectively, produced. SOC appeared to be the least favorable growth substrate for MnP secretion, while fermentation in presence of banana peels and wheat straw provided the highest MnP activity of 240 U/L after four and seven days of cultivation, respectively. Among seven lignocellulosic materials tested, mandarin peels appeared to be the best growth substrate for LiP accumulation; this enzyme activity 2- to 5-fold exceeded those in the presence of other substrates. It is worth noting that fungal growth in all media was visually equal and significant levels of cellulase were produced to ensure culture with carbon and energy source (Table 3).

**Figure 1:**
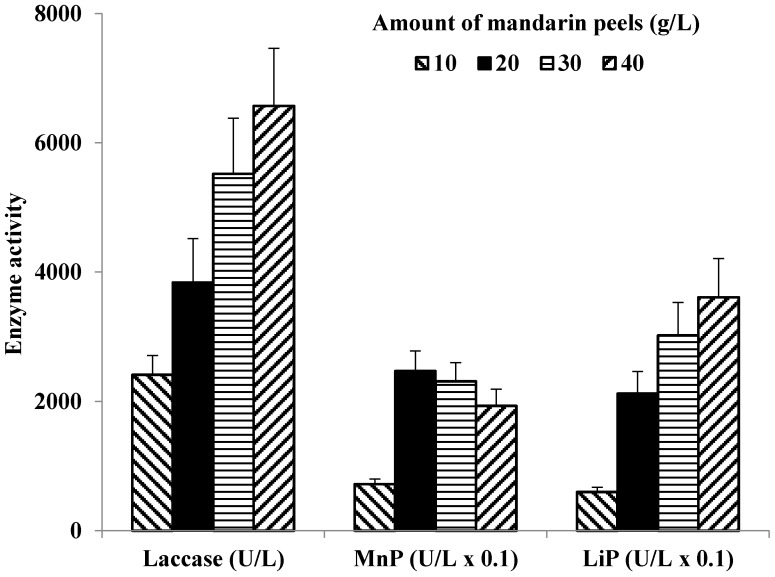
The effect of amount of mandarin peels in the cultivation medium on *C. gallica* 142 LME activities. The synthetic medium containing 10 g/L glycerol was supplemented with mandarin peels at various concentration.

To determine optimal concentration of mandarin peels for LiP production, four concentrations were tested in growth medium. With a gradual increase of the mandarin peels content (from 10 to 40 g/L), the level of *C. gallica* LiP activity increased up to 6-fold, from 600 to 3610 U/L (Figure 1). Similarly, the laccase activity increased and almost tripled from 2410 U/L when 10 g/L of mandarin peels concentration was used to 6570 U/L when 40 g/L of mandarin peels was added into the growth medium. *C. gallica* 142 MnP activity showed an increase from 720 U/mL to 2470 U/L when mandarin peel concentration was changed from 10 to 20 g/L. However, further increase in concentration of growth substrate caused a statistically insignificant decrease in MnP activity.

**Figure 2:**
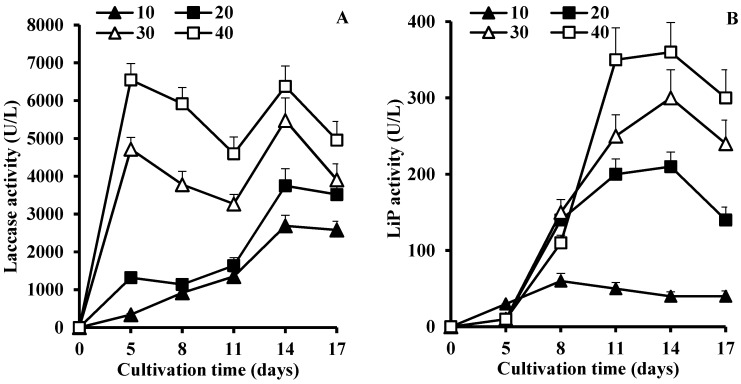
Profiles of laccase (**A**) and LiP (**B**) activities of *C. gallica* 142 in cultivation media supplemented with varied concentration of mandarin peels (g/L).

Production of LME activities differed significantly dependent on the mandarin peels concentrations (Figure 2). For example, laccase production during the first five days of *C. gallica* cultivation (Figure 2A) was significantly enhanced (19-fold) with a parallel increase in mandarin peels concentration. Moreover, at lower substrate concentration, laccase activity gradually increased while two peaks reflecting laccase activity were present when 30 g/L and 40 g/L of mandarin peels were used. Then, activity of LiP increased gradually in all media peaking during the eighth day of cultivation with 10 g/L of mandarin peels and on the fourteenth day with 30 and 40 g/L of substrate (Figure 2B).

### 3.3. Effect of Nitrogen Source and Surfactant Concentration on LME Production

**Figure 3:**
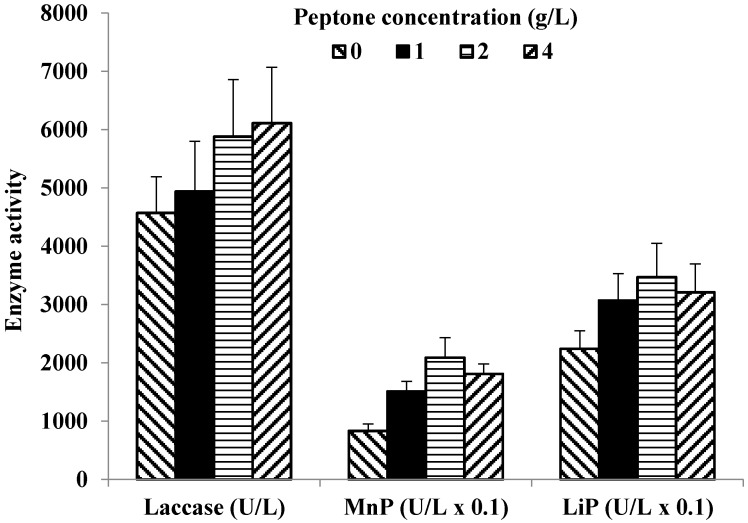
The effect of peptone concentration on the *C. gallica* 142 LME activities. The nutrient medium containing 10 g/L glycerol and 40 g/L mandarin peels; peptone concentration varied from 0 to 4 g/L.

To facilitate optimum growth conditions for *C. gallica* 142 culture, peptone concentrations were investigated in relation to the LME production. The experiment results show that laccase production increased from 4570 U/L to 6110 U/L when peptone concentrations increased from 0 to 4 g/L (Figure 3), suggesting a direct correlation with higher biomass production. However, when MnP activity was analyzed, a 3-fold increase in the enzyme activity at peptone concentration of 2 g/L was observed in comparison with the control medium, suggesting possible peptone-based induction. Additional increase in nitrogen concentration (>2 g/L) showed inhibition in MnP secretion. Similar correlation was observed for LiP production: when peptone concentration increased from 0 to 2 g/L, the maximum enzyme activity changed from 220 to 350 U/L. In the subsequent experiments, 2 g/L concentration of peptone was used as a nitrogen source.

LME secretion may be influenced by presence and concentration of variety of surfactants. Addition of Tween 80 to *C. gallica* 142 culture did not significantly affect laccase, LiP or endoglucanase secretion (Table 4) but almost doubled the MnP activity when added at the concentration of 4 g/L. While PEG did not affect *C. gallica* enzyme secretion at any concentration tested, even low concentrations of Triton X-100 caused a 5-fold decrease in MnP activity and inhibited cellulase production. It is worth noting that, during the first five days of cultivation, Triton X-100 completely inhibited MnP and LiP production and caused an 8-fold decrease in laccase production. However, after the initial halt in LME production, increased enzyme synthesis was observed.

### 3.4. Effect of Aromatic Compounds

Supplementation of nutrient medium with aromatic/phenolic compounds seems to be one of the most effective approaches to increase production of LME by WRB. Several well-known LME synthesis modulators were tested in this study. Among the tested compounds, pyrogallol and veratryl alcohol caused a two-fold increase in *C. gallica* 142 laccase activity while supplementation of the medium with ferulic or vanillic acids showed only a slight increase in their secretion (Table 5). None of the tested compounds affected MnP expression and only the presence of pyrogallol showed a 30% increase in LiP activity. Thus, 0.5 mM pyrogallol was used in *C. gallica* cultivation in fermenter.

### 3.5. Enzyme Production in a Laboratory Bioreactor

Evaluation of LiP production and scale up of *C. gallica* 142 culture were performed in a fermenter setting in the optimized mandarin peels-based medium. During the first five days, *C. gallica* 142 was cultivated at pH 5 to provide optimal conditions for polysaccharide hydrolysis and a steady supply of carbon source for the fungal growth. During the next five days, the medium pH was controlled at 5.7 to slow polysaccharide hydrolysis and limit the carbon source as well as to prevent a possible enzyme inactivation driven by higher pH conditions. Simultaneously, the agitation speed was decreased to 200 rpm to diminish the shear force effect on fungal hyphae and enzyme production.

**Figure 4:**
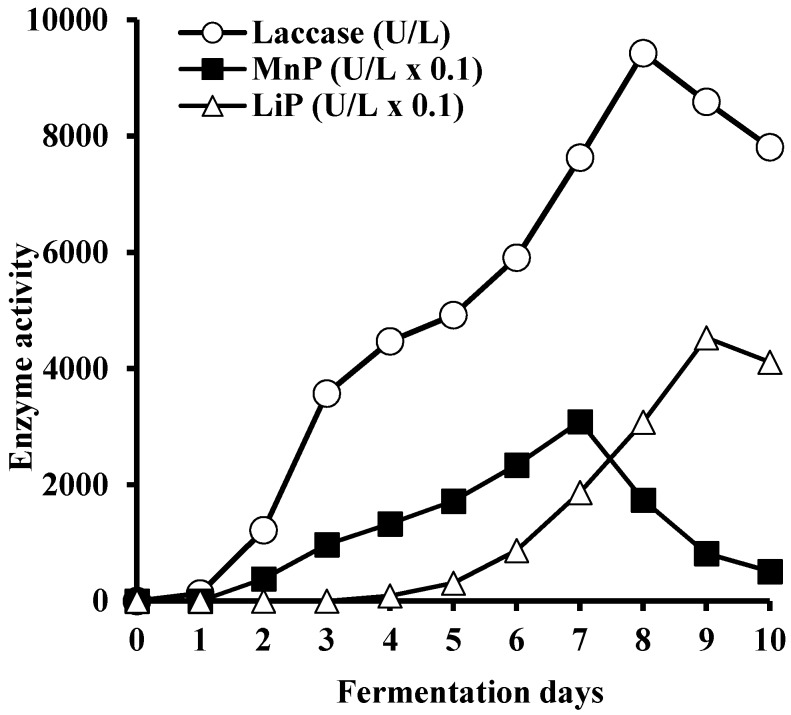
LME activities during cultivation of *C. gallica* 142 in bioreactor.

As shown in Figure 4, the presence of laccase activity was detected after the first day of fermentation, with a gradual increase in its activity throughout the cultivation time, reaching the maximum on day eight (9430 U/L). A low amount of MnP was released during the second day of fermentation with the maximum activity detected on day seven (310 U/L) followed by the sharp decrease through the reminder of cultivation time. LiP activity was detected during the fourth day of fermentation, reaching its maximum (450 U/mL) after nine days of cultivation. The final isolated and concentrated (100 mL) enzyme preparation from *C. gallica* 142 contained 263 U/mL laccase, 2 U/mL MnP and 16 U/mL LiP.

## 4. Discussion

The ligninolytic extracellular enzyme system of WRB is well known to degrade hazardous chemicals and to be utilized in a variety of industrial applications [4]. The large scale production of these enzymes is of great importance. Obviously, there is a large inherent variability within fungal species, with respect to carbon to nitrogen requirements for efficient production of LME [5,8,10,14]. Thus, two substrates, glycerol and mandarin peels, which are known to promote fungal growth and accelerate enzyme secretion [5,9], were added into the basal medium and tested with sixteen white rot fungal strains. To enhance LiP synthesis, the basal medium was supplemented with 0.3 mM veratryl alcohol [2,3,15]. Fungal growth in the tested medium resulted in production of an array of LME concentrations across fungal species (Table 1 and Table 2).

The data received clearly indicate that the tested fungal strains display a wide intra- and interspecies diversity in their ability to produce LME in the synthetic medium. Four *C. unicolor* strains, *T. zonatus* 540 and *T. versicolor* 159 secreted high laccase activity (2350 U/L to 9410 U/L) in synthetic glycerol-containing medium (Table 1). In the other studies, *C. unicolor* C-137 also accumulated 4000 U/L laccase activity in the synthetic medium with glucose [26]. In contrast, only trace amounts of this enzyme were detected in cultivation of *P. radiata* and *T. ochracea*, like *Ganoderma* spp., *Pleurotus tuber*-*regium* [5,14] and *Pycnoporus coccineus* [4]. Moreover, only *C. unicolor* expressed comparatively high MnP activity in the synthetic medium, which is in agreement with observations of Hibi et al. [27], who showed that *Cerrena* sp. was capable of secreting laccase and three peroxidases in submerged cultivation in a glucose-containing medium. In contrast, Michniewicz et al. [26] revealed that *C. unicolor* C-137 did not secrete peroxidase activity in both glucose-containing synthetic and in complex tomato juice-based media.

The present study highlights the role of lignocellulosic growth substrate in the LME activity expression. Supplementation of glycerol-containing medium with mandarin peels caused an overall increase in laccase activity for all screened WRB (Table 2). It is worth noting that, in several cultures, such as *F. trogii* 146, *T. hirsuta* 119, and *T. versicolor* 159, an elevated laccase activity may be explained by an increase in fungal biomass, similarly to other studies [8,14]. However, for cultures such as *C. unicolor* 301, *M. tremellosus* 206, *P. radiata* 64658 and *T. ochracea* 1009, this increase in laccase activity occurred due to induction in the presence of mandarin peels. Moreover, mandarin peels stimulated the MnP secretion by individual WRB and provided induction of this enzyme synthesis by *M. tremellosus* 206 and several other fungi. The enhanced/induced MnP production during cultivation on this substrate may be attributed to the pool of water-soluble aromatic compounds as well as flavonoids present in the mandarin peels [25] and release into the nutrient medium during sterilization and the substrate degradation. These observations suggest that the presence of the lignocellulosic substrate in the nutrient medium is a prerequisite for MnP production. The results of the presented study are in agreement with earlier findings, which showed a lack of MnP activity during cultivation of fungi in the synthetic media but significant MnP expression in the media with plant materials [13,14]. This study indicates that LiP production was induced in cultures of *C. unicolor*, *F. trogii*, *T. ochracea*, and *T. zonatus* by supplementing the medium with mandarin peels. Up to this date, this is the first evidence of LiP production by these four fungal species. On the contrary, two *P. chrysosporium* strains, well-known for LiP secretion, showed only traces (<0.02 U/mL) of LiP activity in cultivation with mandarin peels. This observation may be explained by either an absence of chemical compounds necessary for enzyme expression in the simple medium used for cultivation or a lack of secondary metabolism phase induced by nitrogen and carbon starvation conditions [2,6,7,16]. It is interesting that, in the comprehensive research, Kinnunen et al. [26] screened 53 species of basidiomycetes for lignin modifying enzymes when cultivated in liquid mineral, soy, peptone and solid state oat husk medium and specified that relatively high LiP activities were obtained in mineral medium under low carbon (5 g/L glucose) and nitrogen (2 mM) conditions. In our study, the basal medium supplemented with mandarin peels is characteristic of high carbon and nitrogen content, and it may inhibit or delay secondary metabolism that triggers LiP synthesis. Therefore, the presented data show that, unlike *P. chrysosporium* and several other WRB, *C. unicolor* strains and *C. gallica* have an ability to synthesize high levels of LME under comparatively high carbon and high nitrogen conditions during an active phase of growth.

The production of LME by *C. gallica* 142 and the ratio of the individual enzymes, similar to other WRB [5,13,14,28] was clearly dependent on the lignocellulosic growth substrate and its concentration (Table 3, Figure 1). For example, sunflower oil cake provided maximum laccase activity of *C. gallica* 142, wheat straw promoted MnP secretion while mandarin peels increased the culture’s LiP expression. The reason by which these materials specifically improve individual enzyme activity is not yet clear. It is evident that these lignocellulosic substrates have different chemical compositions and differ significantly in aromatic compounds content that may be released to the liquid medium during sterilization and fungal growth. It is also possible that new aromatic compounds appeared during lignocellulose metabolism, enriching the pool of new LME inducers. There are only a few comparative studies that summarize differences in fungal secretomes produced in response to the addition of plant-residue based natural phenolic elicitors, and little is known regarding activation of specific LME isoenzymes by these compounds. Therefore, additional studies are required to deepen understanding on the expression and regulation of LME genes in the presence of individual plant-derived inducers.

A number of reports indicate that aromatic/phenolic compounds, especially those structurally related to lignin, play an important role in regulation of the LME production in WRB [2,3,5,13,15,29]. Some studies suggest that specific compounds contribute to expression of LME with the same aromatic compound playing dual roles, inducer or repressor, depending on the fungal species and enzyme tested [6,29]. Thus, addition of hydroquinone to the *T. versicolor* culture caused simultaneous threefold increase in laccase production and twofold decrease in MnP secretion. On the contrary, *C. unicolor* strain showed decreased laccase activity under the same cultivation conditions [29]. MnP and LiP production by *P. chrysosporium* [16] and several other fungi were significantly increased by the addition of veratryl alcohol. However, no effect of veratryl alcohol on the synthesis of LME was shown in this study.

The overall results of this work aid in a better understanding of LME production by analyzed white rot fungi with a specific interest in LiP production by *C. gallica.* In particular, the *C. gallica* enzyme secretion is modulated by individual carbon and nitrogen sources, but it occurs at comparatively high concentrations and in the absence of toxic inducers. Undoubtedly, this fungus is a good candidate for a scale up fermentation and for production of selected LME. However, more detailed information on regulation of each individual LME synthesis by this fungal species is required for the development of cost-effective production and application technologies.

## Figures and Tables

**Table 1:** Screening of WRB for LME production in the synthetic glycerol-containing medium. Fungi were grown in shake flasks for 14 days at 27 °C (*P. chrysosporium* at 37 °C); the nutrient medium contained 10 g/L glycerol as a carbon source and 0.3 g/L veratryl alcohol as an enzyme synthesis inducer.

Fungi	Final pH	Laccase (U/L)	MnP (U/L) (Mn^2+^) ^a^	MnP (U/L) (Phenol Red) ^a^	LiP (U/L)
*C. unicolor* 300	6.1	8810 ± 1270 ^14,b^	1640 ± 300 ^4^	1440 ± 210 ^4^	0
*C. unicolor* 301	5.8	3190 ± 360 ^10^	810 ± 100 ^10^	1350 ± 220 ^10^	Traces ^c^
*C. unicolor* 302	6.0	7640 ± 1370 ^10^	560 ± 150 ^7^	670 ± 110 ^10^	0
*C. unicolor* 303	5.8	9410 ± 1420 ^10^	1040 ± 210 ^7^	880 ± 160 ^10^	Traces
*C. gallica* 142	5.7	1090 ± 150 ^14^	140 ± 20 ^10^	180 ± 20 ^7^	70 ± 10 ^14^
*F. trogii* 146	5.9	640 ± 103 ^7^	40 ± 10 ^10^	0	Traces
*M. tremellosus* 206	5.3	610 ± 71 ^7^	0	0	0
*P. chrysosporium* 1309	6.5	0	110 ± 20 ^7^	40 ± 10 ^7^	Traces
*P. chrysosporium* 24725	6.1	0	0	0	Traces
*P. chrysosporium* 34541	6.4	0	0	0	0
*P. radiata* 64658	6.0	230 ± 40 ^7^	Traces ^c^	0	Traces
*T. hirsuta* 119	5.5	810 ± 120 ^10^	Traces	140 ± 30 ^4^	0
*T. ochracea* 1009	6.0	250 ± 30 ^7^	280 ± 50 ^7^	210 ± 30 ^4^	Traces
*T. versicolor* 113	6.4	1060 ± 170 ^10^	Traces	0	Traces
*T. versicolor* 159	6.4	2350 ± 380 ^4^	100 ± 20 ^4^	90 ± 20 ^4^	0
*T. zonatus* 540	6.5	4220 ± 690 ^4^	160 ± 30 ^4^	150 ± 20 ^4^	Traces

^a^ MnP substrates; ^b^ the numbers indicate the days of peak activity; ^c^ activities lower than 0.02 U/mL.

**Table 2:** Screening of WRB for LME production in the glycerol + mandarin peels-containing medium. Fungi were grown in shake flasks for 14 days at 27 °C (*P. chrysosporium* at 37 °C); the nutrient medium contained 10 g/L glycerol and 20 g/L mandarin peels as carbon sources and 0.3 g/L veratryl alcohol as an enzyme synthesis inducer.

Fungal Strain	Final pH	Laccase (U/L)	MnP (U/L) (Mn^2+^) ^a^	MnP (U/L) (Phenol Red) ^a^	LiP (U/L)
*C. unicolor* 300	5.8	19,600 ± 3020 ^10,b^	1980 ± 370 ^4^	820 ± 130 ^4^	160 ± 20 ^10^
*C. unicolor* 301	5.0	16,600 ± 2810 ^7^	2760 ± 420 ^7^	1120 ± 180 ^7^	110 ± 20 ^14^
*C. unicolor* 302	6.0	22,430 ± 2720 ^10^	430 ± 40 ^7^	330 ± 60 ^7^	100 ± 30 ^10^
*C. unicolor* 303	5.2	38,290 ± 6080 ^7^	1920 ± 280 ^4^	1070 ± 220 ^7^	60 ± 10 ^10^
*C. gallica* 142	5.2	5300 ± 780 ^10^	170 ± 30 ^7^	220 ± 30 ^14^	210 ± 30 ^14^
*F. trogii* 146	6.0	1200 ± 130 ^4^	90 ± 10 ^10^	270 ± 50 ^10^	60 ± 10 ^14^
*M. tremellosus* 206	4.9	3900 ± 470 ^14^	70 ± 10 ^10^	60 ± 10 ^10^	0
*P. chrysosporium* 1309	6.7	0	160 ± 20 ^4^	0	150 ± 30 ^10^
*P. chrysosporium* 24725	6.2	0	150 ± 30^4^	0	Traces ^c^
*P. chrysosporium* 34541	6.2	0	Traces ^c^	0	Traces
*P. radiata* 64658	5.1	4200 ± 640 ^7^	240 ± 30 ^4^	50 ± 10 ^4^	40 ± 10 ^10^
*T. hirsuta* 119	4.8	1300 ± 190 ^4^	30 ± 0 ^4^	0	30 ± 10 ^4^
*T. ochracea* 1009	5.6	5530 ± 1070 ^4^	380 ± 70 ^4^	170 ± 30 ^10^	80 ± 10 ^14^
*T. versicolor* 113	5.7	3180 ± 390 ^10^	50 ± 10 ^10^	80 ± 10 ^10^	110 ± 20 ^4^
*T. versicolor* 159	6.0	3920 ± 480 ^10^	Traces	0	40 ± 10 ^10^
*T. zonatus* 540	5.7	8430 ± 1390 ^4^	220 ± 40^7^	130 ± 20 ^4^	60 ± 10 ^14^

^a^ MnP substrates; ^b^ the numbers indicate the days of peak activity; ^c^ activities lower than 0.02 U/mL.

**Table 3:** The effect of lignocellulosic growth substrates on the *C. gallica* LME and endoglucanase activity. The fungus cultivation was performed in the nutrient medium containing 10 g/L glycerol and 20 g/L lignocellulosic materials as a carbon source; no inducer was added.

Growth Substrate	Final pH	Laccase (U/L)	MnP (U/L) (Mn^2+^) ^a^	MnP (U/L) (Phenol Red) ^a^	LiP (U/L)	CMCase (U/mL)
Banana peels	5.0	7120 ± 1470 ^7,b^	240 ± 30 ^4^	200 ± 20 ^7^	70 ± 20 ^10^	1.6 ± 0.2 ^7^
EPR	5.4	6130 ± 990 ^7^	110 ± 20 ^7^	260 ± 30 ^7^	40 ± 10 ^10^	2.7 ± 0.2 ^10^
Mandarin peels	5.0	6490 ± 820 ^14^	160 ± 20 ^7^	240 ± 30 ^10^	230 ± 30 ^10^	5.2 ± 0.8 ^10^
Sunflower oil cake	6.0	27,280 ± 4970 ^10^	Traces ^c^	170 ± 50 ^7^	80 ± 10 ^7^	3.5 ± 0.5 ^10^
Walnut pericarp	5.8	4680 ± 610 ^7^	Traces	180 ± 30 ^7^	60 ± 10 ^10^	2.1 ± 0.3 ^7^
Wheat bran	5.0	19,720 ± 2790 ^7^	170 ± 30 ^7^	260 ± 40 ^7^	60 ± 10 ^10^	4.1 ± 0.5 ^7^
Wheat straw	6.4	11,210 ± 1580 ^7^	240 ± 10 ^7^	330 ± 50 ^7^	90 ± 10 ^7^	3.5 ± 0.4 ^7^

^a^ MnP substrates; ^b^ the numbers indicate the days of peak activity; ^c^ activities lower than 0.02 U/mL.

**Table 4:** The effect of addition of surfactants on LME and CMCase production by *C. gallica* 142 in submerged fermentation of mandarin peels.

Surfactant Concentration (g/L)	Final pH	Laccase (U/L)	MnP (U/L) (Phenol Red)	LiP (U/L)	CMCase (U/mL)
Control	5.8	6680 ± 820 ^14^	230 ± 30 ^14^	340 ± 50 ^14^	6.0 ± 0.8 ^14^
+1.0 Tween 80	5.8	6830 ± 1060 ^14^	310 ± 40 ^14^	370 ± 40 ^14^	6.5 ± 1.0 ^14^
+2.0 Tween 80	5.8	6630 ± 1150 ^14^	350 ± 50 ^14^	360 ± 50 ^14^	6.9 ± 1.0 ^14^
+4.0 Tween 80	5.8	7120 ± 1070 ^14^	450 ± 70 ^14^	370 ± 60 ^14^	6.6 ± 1.1 ^14^
+2.0 PEG	5.8	6390 ± 1180 ^14^	220 ± 30 ^14^	340 ± 40 ^14^	6.6 ± 0.9 ^14^
+4.0 PEG	5.8	6720 ± 930 ^14^	230 ± 30 ^14^	370 ± 40 ^14^	6.7 ± 1.1 ^14^
+6.0 PEG	5.8	6900 ± 760 ^14^	220 ± 40 ^14^	370 ± 50 ^14^	6.9 ± 1.2 ^14^
+1.0 Triton X-100	5.1	6310 ± 980 ^14^	40 ± 10 ^14^	400 ± 60 ^14^	4.0 ± 0.5 ^14^
+2.0 Triton X-100	5.0	6370 ± 1190 ^14^	50 ± 10 ^14^	400 ± 70 ^14^	4.3 ± 0.2 ^14^
+4.0 Triton X-100	4.9	6630 ± 1410 ^14^	40 ± 10 ^14^	390 ± 70 ^14^	3.5 ± 0.2 ^14^

**Table 5:** The effect of addition of aromatic compounds on LME production by *C. gallica* 142 in submerged fermentation with mandarin peels.

Compound Concentration (mol/L)	Final pH	Laccase (U/L)	MnP (U/L) (Phenol Red)	LiP (U/L)
Control	5.8	6500 ± 90 ^9^	260 ± 30 ^12^	330 ± 40 ^12^
+0.3 mM hydroquinone	5.2	4920 ± 110 ^12^	240 ± 30 ^12^	140 ± 30 ^12^
+0.3 mM trinitrotoluene	5.8	5320 ± 100 ^6^	210 ± 30 ^12^	290 ± 60 ^12^
+0.5 mM 2.6 dimethoxyphenol	5.6	6190 ± 70 ^6^	220 ± 20 ^12^	370 ± 60 ^12^
+0.5 mM ferulic acid	5.5	9730 ± 1680 ^12^	240 ± 20 ^12^	300 ± 40 ^12^
+0.5 mM guaiacol	5.5	6780 ± 970 ^12^	270 ± 30 ^12^	370 ± 40 ^12^
+0.5 mM pyrogallol	5.5	12,610 ± 1670 ^9^	270 ± 30 ^9^	430 ± 60 ^12^
+0.5 mM vanillic acid	5.5	8330 ± 980 ^12^	240 ± 20 ^12^	330 ± 50 ^12^
+0.5 mM veratryl alcohol	5.3	11,790 ± 1370 ^12^	250 ± 20 ^12^	330 ± 40 ^12^
+0.5 mM xylidine	5.7	7100 ± 1280 ^6^	280 ± 40 ^12^	270 ± 60 ^12^
